# Identification and Quantitative Determination of Lactate Using Optical Spectroscopy—Towards a Noninvasive Tool for Early Recognition of Sepsis †

**DOI:** 10.3390/s20185402

**Published:** 2020-09-21

**Authors:** Karthik Budidha, Mohammad Mamouei, Nystha Baishya, Meha Qassem, Pankaj Vadgama, Panayiotis A. Kyriacou

**Affiliations:** 1 Research Centre for Biomedical Engineering, School of Engineering and Mathematical Sciences, University of London, Northampton Square, London EC1V 0HB, UK; Mohammad.Mamouei@city.ac.uk (M.M.); Nystha.Baishya@city.ac.uk (N.B.); Meha.Qassem@city.ac.uk (M.Q.); p.kyriacou@city.ac.uk (P.A.K.); 2 Interdisciplinary Research Centre (IRC) in Biomedical Materials, Queen Mary University of London (QMUL), Mile End Road, London E1 4NS, UK; p.vadgama@qmul.ac.uk

**Keywords:** near infrared spectroscopy, UV/visible spectra, mid-infrared spectra, blood lactate, sepsis

## Abstract

Uninterrupted monitoring of serum lactate levels is a prerequisite in the critical care of patients prone to sepsis, cardiogenic shock, cardiac arrest, or severe lung disease. Yet there exists no device to continuously measure blood lactate in clinical practice. Optical spectroscopy together with multivariate analysis is proposed as a viable noninvasive tool for estimation of lactate in blood. As an initial step towards this goal, we inspected the plausibility of predicting the concentration of sodium lactate (NaLac) from the UV/visible, near-infrared (NIR), and mid-infrared (MIR) spectra of 37 isotonic phosphate-buffered saline (PBS) samples containing NaLac ranging from 0 to 20 mmol/L. UV/visible (300–800 nm) and NIR (800–2600 nm) spectra of PBS samples were collected using the PerkinElmer Lambda 1050 dual-beam spectrophotometer, while MIR (4000–500 cm^−1^) spectra were collected using the Spectrum two FTIR spectrometer. Absorption bands in the spectra of all three regions were identified and functional groups were assigned. The concentration of lactate in samples was predicted using the Partial Least-Squares (PLS) regression analysis and leave-one-out cross-validation. The regression analysis showed a correlation coefficient (R^2^) of 0.926, 0.977, and 0.992 for UV/visible, NIR, and MIR spectra, respectively, between the predicted and reference samples. The RMSECV of UV/visible, NIR, and MIR spectra was 1.59, 0.89, and 0.49 mmol/L, respectively. The results indicate that optical spectroscopy together with multivariate models can achieve a superior technique in assessing lactate concentrations.

## 1. Introduction

Once considered a simple by-product of anaerobic metabolism, lactate is now recognized as an important intermediate in cellular bioenergetics [1]. Clinically, lactate is now used as a prognostic indicator for hypoperfusion due to its almost proportional production relationship with the presence of oxygen in tissues. Under healthy, resting conditions, anaerobic glycolysis converts approximately 10% of the body’s pyruvate production into lactate. A majority of which produced comes from skeletal muscle (40–50%), the brain (13%), and adipose tissue. In blood, the lactate produced is predominantly due to red blood cells (80%) and leukocytes (13%) [2]. In conditions of inadequate perfusion with low oxygen delivery to the tissues, the anaerobic glycolysis increases. This, in turn, causes a build-up of its by-products, i.e., pyruvate, NADH, and H^+^, and ultimately lactate [3]. Although the liver (20–30%), kidneys (20%), and the myocardium (5–15%) uptake the overproduced lactate, it is a saturable process. This build-up of lactate concentration in blood exceeding a reference value of 2 mmol/L is commonly referred to as hyperlactatemia. A moderate to severe hyperlactatemia with concurrent metabolic acidosis is ascribed as Lactic acidosis (usually >5 mmol/L) [4].

Hyperlactatemia is a common occurrence in critically ill patients during sepsis/septic shock and is considered a powerful predictor of significant morbidity and mortality. An accurate and timely diagnosis of hyperlactatemia by means of measuring blood lactate level is therefore of paramount importance in surviving sepsis [3]. Patients with late diagnosis often suffer from lasting effects such as missing appendages, organ dysfunction like kidney failure or cognitive impairment. Therefore, measuring the serum lactate level in critic care patients has now become a mainstay in assessing the end-organ hypoperfusion and to evaluate the response to resuscitation [5,6,7]. Moreover, measuring the serum lactate level has shown to be beneficial in diagnosing other clinical conditions such as pulmonary embolism, gastrointestinal bleeding and acute abdominal pain [8].

In current clinical practice, the gold standard method to measure blood lactate levels is using an arterial blood gas analyzer (ABG), which requires a sample of arterial blood drawn from the radial artery or a preexisting arterial line. However, these instruments are complex and often require a trained nurse to operate. More importantly, they are invasive, require large volumes of blood (100–200 μL) and provide intermittent measurements [9]. Portable devices which require small sample volume of blood, saliva, or tears to measure lactate are also available in clinical practice. These devices, usually referred to as amperometry and photometry meters, offer quick response time but suffer from single-point measurements and accuracy. Therefore, these devices are not used very widely in critical care and are confined to primary care practice. In the absence of a continuous blood lactate monitor and the slowness of discrete photometric assays, researchers in the past few years have focused on developing ex vivo and in vivo electrochemical lactate sensing systems [9]. Ex vivo sensing systems are commonly used with implanted microdialysis or ultrafiltration probes. In vivo biosensors, on the other hand, are implanted subcutaneously, and provide a direct measurement of the blood lactate concentration. However, these electrochemical sensors are invasive and suffer from poor reproducibility, and are hence still being investigated. More recently, a wearable noninvasive device was developed for detection of lactate in sweat using an organic electrochemical transistor. However, the sensing range of the device is limited to concentrations below 1 mmol/L [10]. Thus, there still subsists an unmet clinical need for noninvasive continuous blood lactate monitor that is easy-to-use, cost-effective, and produces reproducible results.

To address this essential need, the current paper explores the possibility of using optical spectroscopy together with multivariate analysis as a feasible tool for noninvasive prediction of lactate concentration. The proposed methodology is based on the hypothesis that the absorption of light in the visible, near-infrared, or mid-infrared regions is directly or indirectly sensitive to variations in blood lactate levels and would thus show a fluctuation in the absorption spectrum. Nevertheless, these variations in absorption are usually masked by the absorption of other strong absorbents such as water. Therefore, to effectively analyze and quantify the concurrent changes occurring at various wavelengths, and to accurately predict the concentration of lactate based on those systemic changes, the use of multivariate analysis is proposed. A similar approach, previously undertaken by Lafrance et al. in the Near-Infrared (NIR) spectrum (1540–1740 nm and 2050–2400 nm) has resulted in promising yet inconclusive results, and the light interaction with lactate molecules in the entire optical spectrum remains unexplored [11,12]. Consequently, to accomplish the above goal, the initial step of the methodology proposed here is to examine the optical properties of lactate in a solute similar to blood with fewer absorbents, and a large physiological range (0 to 20 mmol/L). Whole blood was not the primary choice as blood contains various chromophores which absorb light significantly in different regions, such as the hemoglobin species (oxy- and deoxyhemoglobin) which absorb light largely between 450 and 1100 nm [13], or glucose, triacetin, and urea which absorb light predominantly in the NIR and MIR regions between 2000 and 2400 nm and 980 and 1200 cm^−1^, respectively [14,15]. To truly investigate and understand the effect of lactate concentration change on the optical spectra, it is important to isolate the absorption by other chromophores. Therefore, a simple blood analogous with osmolarity similar to blood such as Phosphate-Buffered Saline (PBS) is proposed.

The investigations presented in this paper involve the acquisition of optical spectra in the visible/UV, NIR, and MIR regions of the optical spectrum, identification of regions in the acquired spectra where lactate is absorbed and, quantification of lactate concentration from the acquired spectra [16]. The results of the paper are intended to be used as an overlay for spectral detection of lactate in more complicated media such as whole blood or in vivo measurements.

## 2. Materials and Methods

### 2.1. Reagent Preparation

Thirty-seven equivolume samples containing varied concentrations of sodium lactate (NaLac) and isotonic PBS were prepared for this investigation. The concentration of the samples ranged between 0 and 20mmol/L. Isotonic PBS was used because the osmolarity and particle concentration of PBS corresponds to that of the human body. Stock solutions of NaLac and Isotonic PBS were prepared by using analytical grade Sodium L-lactate ($C_{3}H_{5}NaO_{3}$–98+%) (L14500, Alfa Aesar, Lancashire, UK) and PBS 10X (Thermo Fisher Scientific, Waltham, MA, USA) in powder form, respectively. Stock sodium lactate solution of 600 mmol/L was prepared by dissolving 67.236 g of NaLac powder in a liter of deionized water (Pure Klenz, Watford, UK). A liter of aqueous PBS (1X) was made by dissolving 9.89 g of PBS 10x powder in a liter of deionized water. Thirty-seven 30 mL samples with varying concentrations were produced by serially diluting the NaLac stock solution with PBS. The concentration of the first 21 NaLac samples ranged between 0 and 5 mmol/L with an interval of 0.25 mmol/L. In the rest of the samples, the concentration was varied from 5 to 20 mmol/L with steps of 1 mmol/L. The pH (7.4 ± 0.2) and temperature (24 °C ± 0.5) of all 37 samples were kept consistent throughout the measurement period to avoid any artifacts in the spectra. Before obtaining optical spectra, the concentration of all the samples was verified using the LM5 lactate analyzer (Analox Instruments Limited, Amblecote, Stourbridge, UK), while the pH and temperature of all the samples were recorded using the Orion Star A211 Advanced pH Benchtop Meter (Thermo Fisher Scientific, Waltham, MA, USA) [17].

Using the same procedure outlined above, six high concentration (HC) (100, 200, 300, 400, 500, and 600 mmol/L) samples were also prepared. These samples were used to validate and highlight the detected spectral peaks.

### 2.2. Instrumentation

Spectral acquisition of UV/Visible, NIR, and MIR spectra were performed using two different spectrophotometers. The acquisition settings and the devices used are as follows.

#### 2.2.1. UV/Visible and NIR Spectra

The Lambda 1050 dual-beam UV/Vis/NIR spectrophotometer (Perkin Elmer Corp, Waltham, MA, USA) was used to acquire the UV/Visible (300–800 nm) and NIR spectra (800–2600 nm) with a spectral resolution of 1 nm. The spectrophotometer was configured as follows.

- Two light sources—a deuterium lamp and a halogen tungsten lamp—were used to shine light in the wavelength ranges between 300 and 319 nm and 320 and 2600 nm, respectively.
- Three photodetectors, namely, the photomultiplier tube (PMT), the indium gallium arsenide detector (InGaAs), and the lead sulfide detector (PbS), were used to detect the transmitted light photons in the regions between 300 and 860 nm, 800 and 1800 nm, and 1800 and 2600 nm, respectively. Three detectors with specified spectral ranges were used to avoid spectral/detector saturation.
- The slit setting for the InGaAs and PbS detectors were set on “servo mode”, whereby the spectrometer monitors the reference beam energy and adjusts the slits accordingly to avoid saturation of the detectors. A fixed 2 mm slit was used for the PMT detector.
- The gain of the PMT, InGaAs, and PbS detector was set to auto, 5, and 1, respectively, while the response time of all three detectors was set to 0.2 s.
- Prior to the acquisition of NaLac spectra, baseline correction was performed on the spectrophotometer at 100% transmission/0% absorbance to remove background noise. To ensure stable spectral acquisition with high signal-to-noise (SNR), the attenuation in the sample and reference beams were set to 100% and 1%, respectively.

#### 2.2.2. Mir Spectra

MIR spectra were collected with the Spectrum Two Fourier Transform Infrared (FT-IR) Spectrometer (Perkin Elmer Corp, Waltham, MA, USA). The spectral range scanned was from 4000 to 500 cm^−1^ (2500–20000 nm) with a spectral resolution of 1 cm^−1^. The spectrometer was configured as follows.

- The IR source used in the spectrometer is a silicon carbide-based infrared lamp, and the detector used is a deuterated triglycine sulfate (DTGS) detector.
- The Spectrum Two FTIR spectrometer was fitted with a Horizontal Attenuated Total Reflectance (HATR) accessory containing Zince Selenide (ZnSe) crystal trough plate (PIKE Technologies, Madison, WI, USA) instead of a constant path transmission liquid cell. This was to increase the maximum sensitivity of low concentration components through internal reflections in the crystal. The ZnSe crystal is 4 mm thick, 80 mm in length, and has a refractive index of 2.4. The effective angle of incidence is 45°, thus producing 10 reflections in the crystal.
- Background spectrum without a sample was acquired every 20 minutes or between every three samples, whichever came first to remove instrumental and atmospheric contributions to the spectrum of a sample. The HATR accessory was removed while acquiring the background spectra.

### 2.3. Experimental Procedure

#### 2.3.1. Acquisition of UV/Visible and NIR Spectra

Three milliliters of each NaLac sample was transferred into a glass macro cell (Hellma GmbH & Co.KG, Jena, Germany) with an optical path-length of 10 mm. The cuvette containing the sample and an identical blank cuvette was inserted into the sample and reference compartments of the Lamda 1050 spectrophotometer respectively. Three spectra of each sample were acquired in the desired wavelength range (300–1000 nm) and averaged. The resulting spectrum from each sample was considered for further analysis. The same procedure was repeated again with a 300 μL sample in a macro quartz cuvette (Hellma GmbH & Co.KG, Jena, Germany) with a path length of 1 mm to acquire the NIR spectrum (800–2600 nm).

#### 2.3.2. Acquisition of MIR Spectra

The MIR spectra were acquired using 70 μL of each NaLac sample evenly and directly deposited on the ZnSe crystal plate of the HATR Spectrum Two spectrometer. One hundred interferogram scans of each sample were acquired with a scan speed of 0.2 cm s^−1^ and averaged in order to get the MIR spectrum. To prevent any measurement bias, the test samples were chosen at random during spectral collection.

### 2.4. Analysis of Spectra

Once the raw spectra were acquired using the UVWinlab and Spectrum 10 software (Perkin Elmer Corp, Waltham, MA, USA) that accompany the Lambda 1050 and Spectrum Two spectrometers respectively, it was preprocessed and analyzed in MATLAB R2018a (The Math Works Inc., Natic, MA, USA). The first step in preprocessing was to visualize the changes in spectra caused by varying NaLac concentrations by performing baseline spectral subtraction. Whereby, the spectra of the sample with base NaLac concentration (0 mmol/L) was deducted from all the other spectra. This allows for the suppression of spectral features that are relevant to large absorbents such as water and highlight the changes in small spectral peaks. This process also highlighted any large outliers. Following this, the spectra were corrected for any instrument offsets using Robust Linear Multiplicative Scatter Correction (RLMSC). This process emphasizes the small variations in the spectra caused by varying NaLac concentration. The spectra were then filtered and smoothed to remove instrumental high-frequency noise and enhance signal properties using Savitzky–Golay (SG) filter. The polynomial order and the derivative order of the SG filter used to filter spectra from all regions was 2 and 1, respectively. The window length of the filter used for UV/Visible, NIR and MIR spectra was 21, 51, and 31, respectively. Following these steps, any outliers in the data were removed. The same techniques were used to visualize the high concentration samples.

To explicitly extract lactate data from the acquired spectra in the presence of other chemical interferents such as water, sodium, and potassium chloride, and quantitatively predict the concentration of NaLac from the spectra, Partial Least Squares (PLS) regression analysis was used. PLS regression is a multivariate method used to predict a set of dependent variables from a set of independent variables, assuming a linear relationship between the two. The method extracts latent vectors or variables (LV) to model the spectral features that correlate with the analyte concentrations [18]. Each LV describes the extent of covariance in descending order between the calibration spectrum and the concentrations of the analyte. The LVs reduce the dimensionality of the data. The ideal number of LVs required for accurate estimation of lactate concentrations was determined using the Prediction Error Sum of Squares (PRESS) [19].

The PLS model developed was validated using the leave-one-out cross-validation (LOOCV) routine. LOOCV measures how accurately the PLS model will perform in practice. In the LOOCV routine, a randomly selected spectrum of unknown concentration is used as a validation data set and the rest of the spectra are used to train the model. The trained model was then used to predict the concentration of the validation data set. This process was repeated 37 times until every spectrum has been used as a test subject and its concentration was estimated. The same procedure was applied for spectra acquired from all three wavelength ranges, i.e., the UV/Visible, NIR, and MIR. The prediction accuracy of the calibration model was then described by the coefficient of determination (R^2^) between the estimated and actual lactate concentration, and by the estimated measurement error, which was calculated as the root mean squared error of cross-validation (RMSECV).

## 3. Results

### 3.1. UV/Visible Spectra

**Figure 1:**
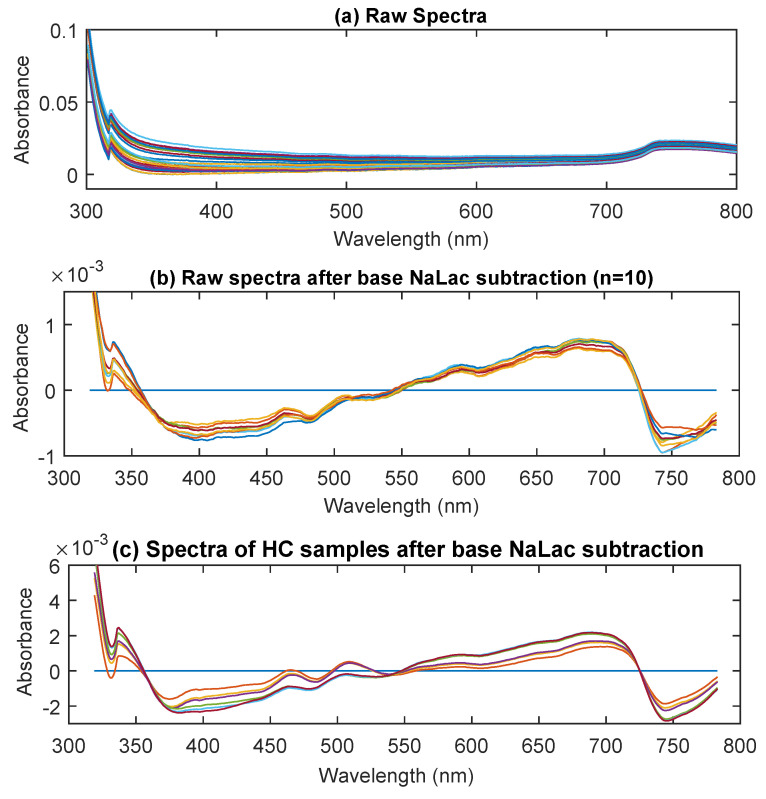
The (**a**) raw UV/Visible spectra of 37 samples with varying concentration of NaLac, (**b**) the raw spectra after base NaLac sample subtraction (0 mmol/l), and (**c**) base subtracted spectra of high concentration samples (100, 200, 300, 400, 500, and 600 mmol/L).

Figure 1a shows the raw absorption spectra of 37 NaLac samples from the UV/Visible region. The absorption in this region is predominantly between 300 to 350 nm and 700 to 800 nm with a distinct peak at 742 nm. In the rest of the visible region between 400 and 700 nm, the absorption is minimal. Upon deduction of the base NaLac (0 mmol/L) spectra from the rest of samples, a few smaller peaks become evident in the 400 nm to 700 nm region (Figure 1b). These absorption bands are at 401 nm, 449 nm, 485, 513 nm, 605 nm, 662 nm, and 742 nm. Note that these peaks are inverted due to the baseline subtraction, which indicates their presence even in samples with no lactate (i.e., absorption due to constituents of isotonic PBS, mostly water). The identified absorption peaks were then assigned to vibrational bands. All the identified peaks are recognized as being different harmonics of water (O-H bonds). The peaks at 742 nm and 662 nm are the 4th harmonic of water and results from O-H bond stretching ($v_{1}/v_{3}$) and O-H stretching and bending ($v_{3}$), respectively. The peak at 605 nm is the 5th harmonic of O-H stretch. The peak at 513 and 485 nm are the 6th harmonic of water and results from O-H bond stretching ($v_{1}/v_{3}$) and O-H stretching and bending ($v_{3}$), respectively. Finally, the peak at 401 nm is 8th harmonic of O-H stretching [20]. To verify the presence of identified peaks, spectra of HC samples were also acquired and are shown in Figure 1c. As can be seen from the figure, the identified peaks are also present in the HC sample set and are more pronounced due to the high concentrations. Although most of the UV/Visible spectra are absorbed by water, there exists a small absorption band at 747 nm, which is relevant to the concentration of lactate [21]. However, this peak is not visible in the spectra, due to the high absorption of water.

**Figure 2:**
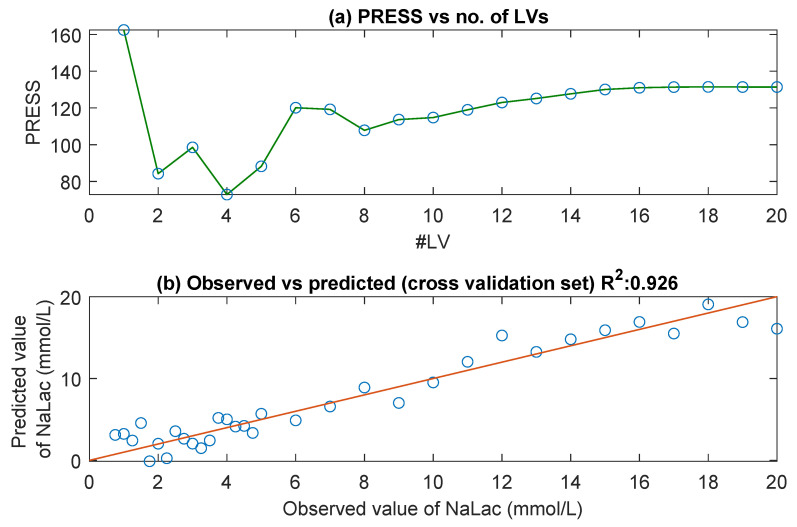
Depicts (**a**) the graph of Prediction Error Sum of Squares (PRESS) versus the number of latent variables (LVs) for UV/visible spectra. The optimal number of LVs used for building the PLS model was two, and (**b**) predicted NaLac concentration vs. the reference NaLac concentration of thirty-seven PBS samples. The correlation coefficient (R^2^) and root mean square error of cross-validation (RMSECV) were 0.926 and 1.59 mmol/L, respectively.

To examine the underlying relationship between the detected absorption peaks and NaLac concentrations and to explore the possibility of accurately predicting NaLac concentrations from UV/Visible spectra, a PLS calibration model was created. The minimum number of LVs required for accurate prediction was determined using PRESS. Figure 2a shows the chart of PRESS vs. the number of latent variables. From the figure, it is evident that the first two LVs contribute the most to the PLS model. From the 3rd LV, it is unlikely that the addition of any more LVs will significantly improve the prediction of NaLac from the spectra. Figure 2b shows the plot of reference NaLac concentrations versus the predicted NaLac concentrations in the leave-one-out routine, using two PLS factors. The diagonal line in the figure represents a good match between the measured and predicted NaLac values. The coefficient of determination (R^2^) and root mean square error of cross-validation (RMSECV) between the predicted and reference values were found to be 0.926 and 1.59 mmol/L.

### 3.2. NIR Spectra

**Figure 3:**
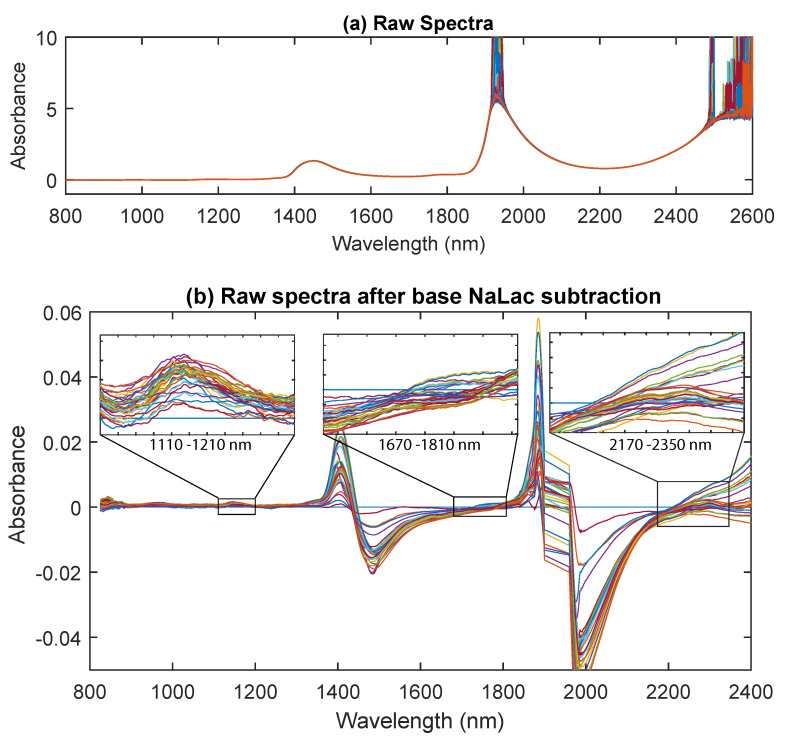
(**a**) The raw near-infrared (NIR) spectra of thirty-seven PBS samples and (**b**) the NIR spectra of samples after base lactate correction. Absorption peaks pertinent to NaLac can be seen in the regions between 1100 and 1210 nm, 1670 and 1810 nm, and 2170 and 23,500 nm.

The raw absorption spectra of thirty-seven PBS samples with varying lactate concentrations between 0 and 20 mmol/L is presented in Figure 3a. As with most NIR spectra, the absorption in the NIR region was dominated by the hydrogen atoms. Two prominent peaks in the spectra can be observed at the 1450 nm and 1920 nm, which can be associated with the O-H stretching fundamental (1st overtone of water) and the combination band of O–H stretch with O–H bend, respectively [22]. Two small peaks at 970 nm and 1180 nm can also be identified, which are associated with the vibrational overtone of the O–H bonds of the water molecules (second overtone) [23,24].

**Figure 4:**
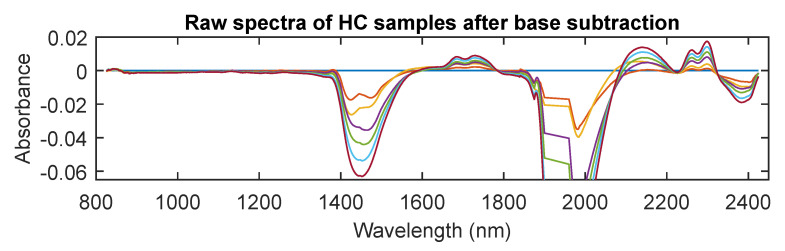
NIR spectra of high concentration samples after base lactate correction. The peaks identified due to the stretching and bending of C-H bonds are visible at 2259, 2299, 1684, and 1730 nm. The absorption at these peaks is linear with the concentration of NaLac in the sample.

It is, however, essential to understand that these wide absorptions are caused by multiple narrow, overlapping absorptions. Therefore, to visibly see changes in other regions more relevant to lactate molecules, the baseline water absorption (NaLac = 0 mmol/L) was subtracted from the rest of the spectra. The high-frequency noise in the 1900–1960 nm and 2350–2600 nm regions was manually removed before zero NaLac spectral subtraction. This was to remove the noise which resulted from detector saturation (due to high absorption of water). Figure 3b shows the spectra of 37 NaLac samples following the base NaLac spectra subtraction. In Figure 3b, an absorption with a magnitude greater than zero theoretically reflects the NaLac absorption, while absorption with magnitude below zero represents water absorption. Although, this is not entirely true in this case due to the small concentration of NaLac (<20 mmol/L). Nevertheless, distinct peaks can now be observed in regions other than the water absorption peaks. These absorption peaks are between 2200 and 2350 nm (centering at 2259 and 2299), 1650 and 1800 nm (centering at 1684 and 1730), and 1200 and 1240 nm (centering at 1215). Absorption in these NIR regions is linked to the combination of C–H stretch with C–H bend (2200–2400 nm) and C-H stretching (1660–1780 nm is the 1st C-H overtone and 1100–1220 nm is the 2nd C-H overtone), respectively [22,25]. To validate the presence of detected peaks, raw spectra from a few high concentration samples were also acquired and is shown in Figure 4. The detected peaks are more distinctly visible in Figure 4 due to the high concentration of the samples, and the concentration of the sample corresponds linearly with the absorbance at the identified peaks [26].

**Figure 5:**
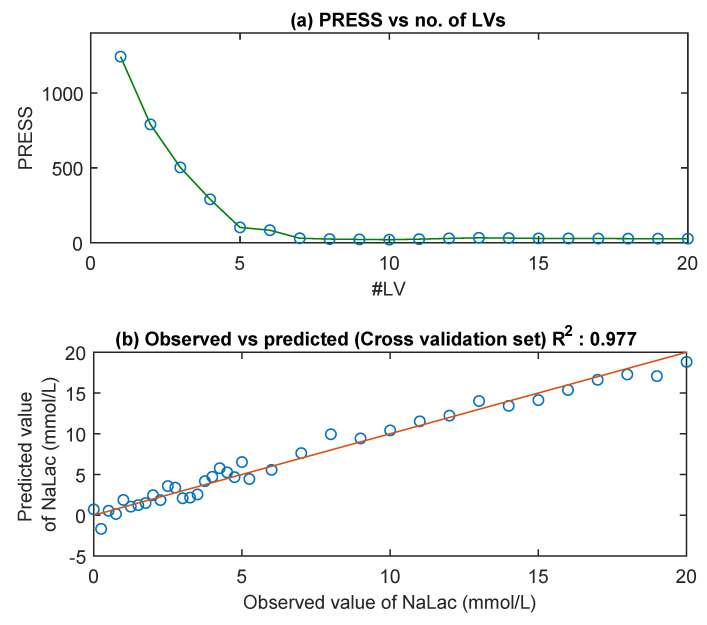
(**a**) The graph of PRESS versus number of latent variables (LV) for NIR spectra. Optimal number of LVs used for building the PLS model were eight, and (**b**) predicted NaLac concentration vs. the reference NaLac concentration of thirty-seven PBS samples. The correlation coefficient (R^2^) and root mean square error of cross-validation (RMSECV) were 0.977 and 0.89 mmol/L, respectively.

Following the determination of the predominant species in the spectra, PLS regression analysis was made on the preprocessed data. Before running the PLS routine, SG derivation was applied to all the spectra. The minimum number of LVs required for an accurate prediction of NaLac concentration was determined using the PRESS. This criterion helps avoid overfitting. Figure 5a shows the graph of predicted residual sum of squares vs. the number of latent variables for NIR data. From Figure 5a, it can be observed that after the 8th LV, it is unlikely that the addition of more LVs will significantly improve the PLS prediction. Therefore, eight LVs were used to build the PLS model. Figure 5b shows the reference vs. predicted NaLac concentration plot, where the predicted values are based on eight PLS factors. A diagonal line, as in the figure usually signifies a great match between the reference and predicted NaLac concentration. The high correlation between the predicted values and the reference values resulted in a coefficient of determination, R^2^, of 0.976 and RMSECV of 0.89 mmol/L.

### 3.3. MIR Spectra

**Figure 6:**
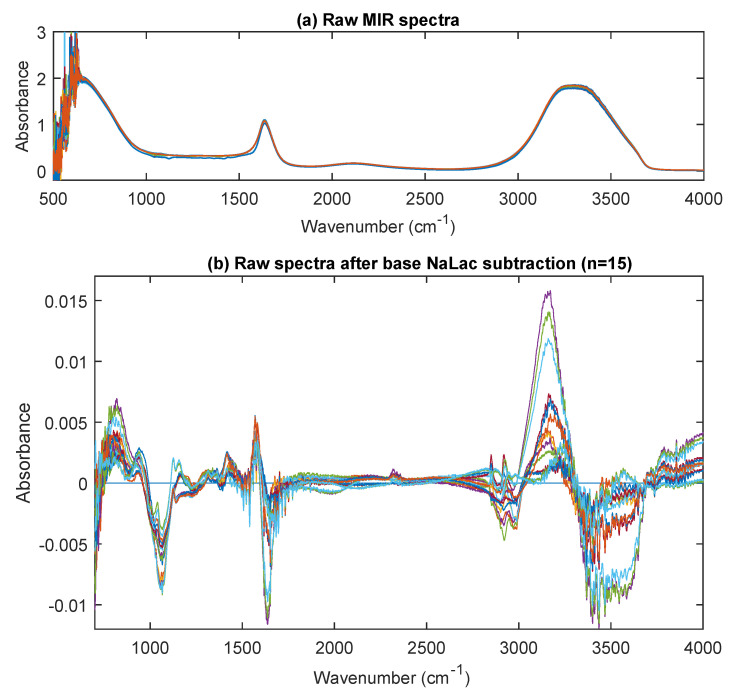
(**a**) Raw MIR spectra (500–4000 cm^−1^) of PBS samples (concentration range: 0–20 mmol/L) (**b**) the raw spectra after noise removal and base lactate correction (n = 15). Several peaks were identified in the footprint region (700–1500 cm^−1^).

Figure 6a shows the raw spectra of 37 NaLac samples (0–20 mmol/L) acquired using the FTIR spectrometer. The spectra provide composite information of all the components in the NaLac PBS medium including lactate, NaCl, KCl and other salts. Two distinct peaks can be seen in Figure 6a, in the diagnostic region of the IR spectrum between 1550 and 1700 cm^−1^ (centering at 1638 cm^−1^), and 3000 and 3700 cm^−1^ (centering at 3283 cm^−1^). The broad peak ranging between 3000 and 3700 cm^−1^ is due to the strong O–H stretching of water in the sample. The narrow peak in the 1550–1700 region is the C=O stretching band and is characteristic of carboxylic acid groups.

Figure 6b shows the same spectra after baseline (0 mmol/L) subtraction. The high-frequency noise peaks in the 500–700 cm^−1^ region were removed manually before the base spectral subtraction. This was to reduce the estimated errors in further analysis. As can be seen from Figure 6b, more bands relevant to the absorption of the NaLac in the solution are visible from the spectra in both the diagnostic region (1500–4000 cm^−1^) and the fingerprint regions (up to 1500 cm^−1^). By identifying the peaks in spectra and matching the frequency with the chemical groups that absorb in MIR region, functional groups were assigned to the corresponding vibrational peaks [27].

**Figure 7:**
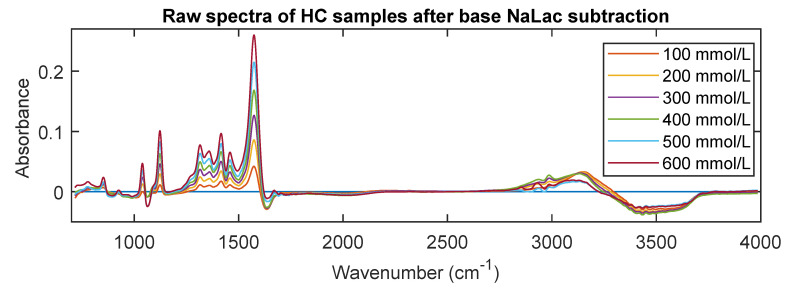
MIR spectra of high concentration samples after base NaLac subtraction. The absorption in each spectrum at the identified peaks linearly proportional to concentration.

Spectra in the fingerprint region showed many overlapping absorbance bands. From Figure 6b, absorption bands were identified at 779 cm^−1^, 854 cm^−1^, 940 cm^−1^, 1040 cm^−1^, 1126 cm^−1^, 1157 cm^−1^, 1315 cm^−1^, 1365 cm^−1^, 1416 cm^−1^, and 1455 cm^−1^. The bands around 779 and 854 cm^−1^ are due to C-H bending, while the bands in the region 900–1200 cm^−1^ correspond the stretching modes of C–C, C–O, and C-OH functional groups [28,29,30]. The absorption in the 1300–1450 cm^−1^ region represents medium CH_3_ stretching. The diagnostic region showed absorbance bands at 2852 cm^−1^, 2930 cm^−1^, 2988 cm^−1^, and 3159 cm^−1^. The bands at 2852, 2930, and 2988 cm^−1^ were assigned to C-H stretching, symmetric stretching of CH_3_, and asymmetric stretching of CH_3_, respectively [31,32,33]. Finally as mentioned earlier, the bands which were below zero after the baseline subtraction were assigned O-H stretching functional group, as the absorption here is due to water. To make sure the bands were identified appropriately, the spectra of HC samples were acquired and is shown in Figure 7. The figure demonstrates all the spectral bands vividly and the absorbance at each peak increases linearly with an increase in concentration (100 mmol/L to 600 mmol/L in steps of 100 mmol/L).

**Figure 8:**
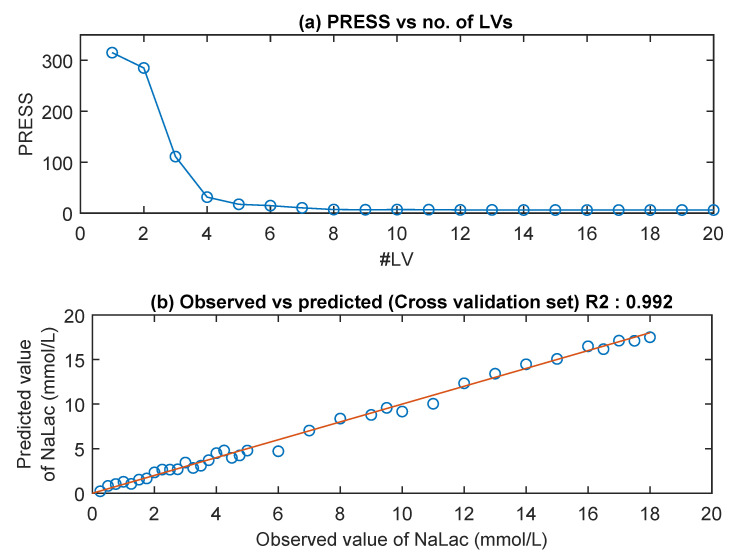
(**a**) PRESS value for the PLS algorithm, optimal no. of LVs used: 8. (**b**) Relationship between the predicted NaLac concentration and the reference NaLac concentration of thirty-seven PBS samples. The correlation coefficient (R^2^) and root mean square error of cross-validation (RMSECV) were 0.992 and 0.49 mmol/L respectively.

Following the determination of the predominant species in the spectra, PLS regression analysis was applied to the preprocessed MIR spectra. As with the UV/Visible and NIR spectra, PRESS was used to determine the number of LVs required for accurate prediction of NaLac concentration from the MIR spectra. Figure 8a shows the graph of PRESS vs. the number of LVs for MIR data. The first eight LVs were chosen to build the PLS model. Figure 8b shows the plot of reference NaLac concentrations and the NaLac concentrations predicted from MIR spectra in the leave-one-out routine. As can be seen from Figure 8b, the linear line shows a great correlation between the predicted and the reference values resulted in a coefficient of determination, R^2^, of 0.992 and RMSECV of 0.495 mmol/L.

## 4. Discussion

Continuous measurement of elevated serum lactate levels in critically ill patients is of paramount importance. A routinely usable clinical tool for noninvasive monitoring of the blood lactate level is still lacking. Time tested techniques such as the intermittent blood gas analysis continue to be the mainstay for determining the serum lactate concentration. Hence, optical spectroscopy combined with multivariate analysis of spectra was proposed in this paper as a pathway towards a noninvasive lactate monitor. As it is essential to first understand the optical properties of lactate in all the regions of the optical spectrum, a rigorous in vitro study was conducted where spectra of varying concentrations of lactate in PBS was acquired in the UV/Visible, NIR, and MIR regions. The study aimed to determine the absorption bands in each region that are pertinent to the absorption of lactate and to predict the concentration of lactate using PLS regression.

Spectra of thirty-seven samples with varying concentration of NaLac ranging between 0 and 20 mmol/L were first acquired in the UV/Visible region. The NaLac samples showed very weak light absorption in the UV/Visible region, which is a common characteristic of clear liquids in this region. Nonetheless, the spectra showed visible absorption bands after postprocessing the spectra at 401 nm, 449 nm, 513 nm 605 nm, and 742 nm (Figure 1). These absorption bands are a sequence of overtones and combination bands of O-H bonds, whose intensity decreases at each step, giving rise to an absolute minimum at 401 nm [20]. Although most of the identified peaks are due to O-H bonds, lactate has been previously reported to absorb at 747 nm [21]. The use of PLS regression routines, to predict the concentration of lactate from the spectra, has resulted in an R^2^ of 0.925 and RMSECV of 1.59 mmol/L. Although a good correlation exists, it might predominantly be linked to the concentration of water and not lactate. This is because the concentration of water reduces with an increase in the concentration of NaLac in equivolume samples, and lactate in aqueous solutions does not contain chromophores that absorb at wavelengths >290 nm. Plus, no absorption bands relevant to any bonds (C-H, CH_3_) other than O-H bonds were identified. Hence, the usability of the UV/Visible spectrum for direct measurement of lactate concentration is marginal. Nevertheless, the UV/visible region could still be very useful in identifying changes in lactate levels indirectly using either the tissue oxygenation status measured from spectra (500–900 nm) or through spectrochemical reactions. The rationale behind this is that an increase in serum lactate is manifested by inadequate perfusion with low oxygen delivery to tissues. Several commercial devices are readily available in the market which make use of these indirect methods. Some of these devices measure tissue oxygenation optically and correlate the tissue oxygen status to lactate concentration [34,35]. Others use the catalyzing effect of lactate oxidase enzyme along with either an electrochemical oxygen sensor [36] or a spectrophotometer [37,38] to build a relationship between lactate concentration and oxygen consumption. Another non-commercial spectrophotometeric method which utilizes UV/Visible region to determine lactate concentration is based on the reaction of lactate ions with iron(III) chloride at 390 nm [39]. The limit of detection for these indirect methods is commonly ≤2.0 mmol/L over the physiological range of 1.0 to 25 mmol/L [40].

The NIR radiation is absorbed mainly by C-H, N-H, and O-H bonds which are primary constituents of organic compounds such as lactate. Therefore, NIR spectra will be very useful in determining the concentration of lactate. However, the peaks in the NIR spectrum are not distinct or sharp because they consist of overtones and combinations from primary absorptions in the mid-infrared regions and also due to light scattering [27,41]. The raw spectra acquired from the samples showed relatively high absorbance compared to the UV/Visible region which was dominated by absorption due to the stretching and bending of O-H bonds. Four absorption bands centred at 970 nm, 1180 nm, 1450 nm, and 1920 nm were identified as overtones and combination bands of O-H bonds [42]. Upon processing the spectra, three regions were identified where the absorption was dominated by C-H bond stretching and bending. These bands were centred at 1215 nm, 1730 nm, 1684 nm, 2299 nm, and 2259 nm. However, the linear changes in absorbance at these bands (O-H and C-H) in the spectra, caused due to the variations in the concentration of lactate are barely noticeable. Therefore, PLS was employed to correlate the changes in NIR spectra to the reference measurements. PLS leave-one-out cross-validation yielded an R^2^ of 0.977 and RMSECV of 0.89 mmol/L. This indicates that although the tight correlation of the data at very low lactate concentrations is not apparent, it is easy to distinguish the changes that are close to 1 mmol/L of lactate in PBS solutions. The results obtained in the NIR region are in close agreement with the previous work conducted by Lafrance et al., where they reported NaLac absorption peaks between 2100–2400 nm [12]. The accuracy of prediction is also comparable or better than that of commercially available hand-held lactate devices such as the Lactate Pro, Lactate Scout and Lactate Plus, which generally produce an error ≤2.0 mmol/L over the physiological range of 1.0 to 18 mmol/L [40,43].

The absorption in the MIR region, as mentioned earlier, is associated with the fundamental vibrations of organic compounds. The MIR spectra showed several fundamental absorption bands in both the diagnostic region and the fingerprint region. In the fingerprint region, many overlapping absorbance bands can be seen at 779 cm^−1^, 854 cm^−1^, 940 cm^−1^, 1040 cm^−1^, 1126 cm^−1^, 1157 cm^−1^, 1315 cm^−1^, 1365 cm^−1^, 1416 cm^−1^, and 1455 cm^−1^. Absorption in these bands is due to intramolecular phenomena and are highly specific to the constituents of NaLac samples. However, due to the presence of several overlapping bands, it is more difficult to assign individual bonds to each peak [44]. Nonetheless, as this region is a single-bond region, the absorption is most likely due to C-C, C-O, C-H, and CH_3_ bonds [28,29,30]. The diagnostic region showed absorbance bands at 2852 cm^−1^, 2930 cm^−1^, 2988 cm^−1^, and 3159 cm^−1^. The absorbance in this region is typically associated with functional groups (e.g., -OH, C=O, CH3, etc.) [31,32]. Due to a large number of peaks and complex shapes of the MIR spectra, the linearity of absorption with an increase in concentration is not visible in the sampling range. This was however evident in the HC spectra. The PLS regression of the MIR spectra resulted in an R^2^ of 0.992 and RMSECV of 0.495 mmol/L, indicating a very high correlation between the predicted and reference values. These results indicate that the prediction accuracy of MIR spectra is comparable to that of the gold standard arterial blood gas analyzer (ABG), which has an accuracy of ≤0.5 mmol/L over the physiological range of 1.0 to 10 mmol/L [45].

It is expected that the accuracy of prediction is the highest in the MIR spectra compared to the NIR and UV/Visible spectra as the absorption in MIR region relates to all the functional groups in the sample solution. However, the use of MIR spectroscopy in measuring blood lactate is limited to in vitro studies, where samples of blood can be examined for the concentration of lactate. In vivo measurements are not possible as the penetration of MIR radiation in human tissue does not go beyond the stratum corneum where no arterial or venous blood is present. Therefore, with the help of fiber-optic ATR-FTIR probes, MIR spectra is currently only used in vivo for distinguishing pre-malignant and malignant tissues [46]. The NIR region, on the other hand, offers relatively good accuracy and can be applied in vivo as the penetration of NIR light is adequate to penetrate the dermal layers where arterial blood is present. Further, considering the fact that the aim of this work is to develop a noninvasive continuous lactate monitor, it is fair to say that any future in vitro and in vivo studies should focus on the NIR spectra.

It is important to notice here that although the prediction accuracy of NIR spectra is comparable to that of OEM devices, the physiological range is quite large (0–20 mmol/L). In critically ill patients suspected with sepsis, the blood lactate level at the time of admission is typically >2 mmol/L and <4 mmol/L. Following a 48-hour ITU admission, a lactate level of 2.25 mmol/L has been identified to be fatal (predictor of mortality with a sensitivity of 72.2 % and specificity of 92.1%) [47]. Therefore, future work will focus on (1) repeated in vitro studies with whole blood and serum as analytes (lactate range: 0–10 mmol/L) to generate a large set of NIR spectra, and (2) improving the multivariate models for better accuracy. The aim here will be to improve the accuracy of prediction using NIR spectra to a similar range as the gold standard ABG, which produces an accuracy of 0.1 mmol/L in the physiological range between 1 and 6 mmol/L [45].

In conclusion, the results of this paper strengthen the hypothesis that the accurate measurement of lactate concentration in buffered solutions is possible using optical spectra and multivariate analysis. The outcomes from this experiment also provide the necessary confidence to complete further investigations in complex solutions with more absorbents such as serum and blood. Specifically, the NIR region seems to provide an optimal window for future noninvasive in vivo evaluations. Future work will concentrate on improving the prediction algorithms utilized in this investigation to quantitatively evaluate NaLac concentrations from the spectra of blood.
